# Serotypes of Aggregatibacter actinomycetemcomitans in relation to periodontal status and geographic origin of individuals-a review of the literature

**DOI:** 10.4317/medoral.19304

**Published:** 2013-12-07

**Authors:** Jandenilson A. Brígido, Virginia R S. da Silveira, Rodrigo O. Rego, Nádia A P. Nogueira

**Affiliations:** 1 Post-graduate Program in Dentistry, Faculty of Pharmacy, Dentistry and Nursing, Federal University of Ceará, Fortaleza, CE, Brazil; 2 Department of Dentistry, School of Dentistry at Sobral, Federal University of Ceará, Sobral, CE, Brazil; 3 Department of Clinical and Toxicological Analyses, Faculty of Pharmacy, Dentistry and Nursing, Federal University of Ceará, Fortaleza, CE, Brazil

## Abstract

Objectives: Several studies have focused on the relationship among serotype distribution, ethnical status and geographic populations, and periodontal conditions. Studies that have investigated the prevalence and the distribution of A. actinomycetemcomitans serotypes and the relation between the different serotypes of the bacterium and periodontal status were reviewed. Material and Methods: A systematic literature search for publications regarding the distribution of A. actinomycetemcomitans serotypes in subgingival samples of periodontitis patients and periodontally healthy subjects by employing polymerase chain reaction (PCR) was conducted. Results: From the 85 studies identified in the first analysis, only 12 met all inclusion and exclusion criteria. Clinical isolates from diverse geographic populations with different periodontal conditions were evaluated. Serotypes a, b and c were largely found, and serotype c was the most prevalent. They were isolated from various periodontal conditions, including aggressive periodontitis. Conclusions: The available literature suggests that serotypes a, b, and c are globally dominant, serotypes d and e are rare, and the prevalence of the most recently identified serotype fis still unknown. It is widely accepted that distribution patterns of A. actinomycetemcomitans vary among subjects of different ethnicity and geographic regions. The correlation of different serotypes with various periodontal conditions remains unclear.

** Key words:**Aggregatibacter actinomycetemcomitans, serotypes, periodontal disease, prevalence.

## Introduction

Periodontitis is a collective term for inflammatory conditions affecting supporting tissues of the teeth induced by microbial deposits (1). Progressive loss of tooth attachment in periodontis may eventually culminate in loss of affected teeth. As a consequence, periodontal disease is one of the most important concerns for dentists, patients and the public dental healthcare system.

Epidemiological studies have shown that periodontal disease occurs predominantly in a slowly progressing form, chronic periodontitis, which in the majority of patients involves a limited number of teeth and rarely interferes with tooth function before adulthood (2). Periodontitis also occurs in a severe and rapidly progressing form, denoted aggressive periodontitis, which most often starts at an early age (2,3).

Clinical and microbiological studies have identified only a few bacterial species associated with periodontal disease in adults (4). *Aggregatibacter actinomycetemcomitans* is a Gram-negative, nonmotile, facultative anaerobic cocobacillus bacterium that colonizes the human oral cavity, associated with the etiology of aggressive periodontitis (5-7), and can also be detected in the oral cavity of chronic periodontitis patients and periodontally healthy subjects (8,9). This microorganism produces a variety of virulence factors, such as lipopolysaccharide, leukotoxin and cytolethal distending toxin (CDT) (10).

Development of techniques to detect the genetic variability of microorganisms has allowed for the observation of genetic differences in the leukotoxin promoter region between various *A. actinomycetemcomitans* strains, which are directly correlated with their leukotoxicity (11). Strains that are highly leukotoxic have a deletion of 530 base pairs in the leukotoxin promoter region, while those that are minimally leukotoxic present contain the complete leukotoxin promoter region. Thus, the highly leukotoxic strains (designated the JP2 clone) can produce 10 to 20-fold more toxin than the others, providing them with the potential to interfere with the host’s innate immune defense (12).

*A. actinomycetemcomitans* can be grouped into six serotypes (a-f) based on the polysaccharide antigen on the cell surface (13). Numerous studies have examined the relationship of *A. actinomycetemcomitans* serotype, ethnical status and geographic populations, and periodontal disease status, but with conflicting results (14-17). Subjects are usually colonized by a single serotype, which can persist for life (18), and the frequency distribution of *A. actinomycetemcomitans serotypes* differs among various populations (19). There are no epidemiological studies on the distribution of *A. actinomycetemcomitans* serotypes, but the available literature suggests that serotypes a, b, and c occur much more frequently among oral isolates than serotypes d, e, and f (13,20-22). Differences in serotype distribution have been shown among African, Asian, Europeans, and North and South American populations (21-25).

The different studies describe different microbiological identification techniques. Detection methods for *A. actinomycetemcomitans* currently used include bacterial culture, DNA probe hybridization (20), specific antibody immunofluorescence (21), gene amplification via PCR methodology (22), including multiplex, nested multiplex and quantitative PCR. Each methodology varies with regard to detection time, the minimum number of cells that can be detected, and the ability to quantify the numbers of *A. actinomycetemcomitans* present in samples. PCR-based methods are not only suitable for the confirmation of strains, but have also been shown to have high sensitivity and specificity for the detection of *A. actinomycetemcomitans* from clinical samples of supragingival and subgingival plaque, and allow rapid detection of *A. actinomycetemcomitans* from clinical samples.

The objective of the present study was to review the studies that have investigated the prevalence and the distribution of *A. actinomycetemcomitans* serotypes in subgingival samples of periodontitis patients and periodontally healthy subjects by employing polymerase chain reaction (PCR) and to examine the possible association between periodontal conditions and serotypes.

## Material and methods

Data sources and search strategy

The electronic database PubMed was searched systematically for studies published between January 2002 and December 2012. No language restrictions were applied. Both Mesh and Major terms were used in the search and Boolean operators (OR, AND) were used to combine the searches. The bibliographies of all potentially relevant studies and review articles were also searched. The search terms included “serotypes” AND “*Aggregatibacter actinomycetemcomitans” OR “Actinobacillus actinomycetemcomitans” AND* “periodontal disease” OR “periodontitis”. The search was carried out twice by two different people.

Study selection

Eligibility criteria applied to all studies retrieved by the search were established. Duplicate records or double-published studies and articles published before 2002 were excluded. No limitations were placed on the geographical location. Studies involving the distribution of *A. actinomycetemcomitans* serotypes in subgingival samples of periodontitis patients and periodontally healthy subjects by employing PCR were eligible for inclusion in this review. All abstracts were reviewed in order to identify any studies of interest. Two reviewers independently assessed the full-text articles for eligibility. Only studies which met all the eligibility criteria were finally included. Relevant data were abstracted from all studies meeting the eligibility criteria. The following data were extracted from each study: (1) the first author and year of publication; (2) the country where the study was conducted; (3) searched serotypes; (4) Aim of study; (5) Principal findings and (6) possible association between periodontal conditions and serotypes.

## Results

Eighty-five articles were identified, of which 66 were excluded based on their titles and abstracts. The full text of each of the 19 remaining papers was reviewed, and seven were excluded because they did not match the inclusion criteria for this review. The remaining 12 studies were included in the review, nine cross-sectional studies and three longitudinal studies.

The study selection is presented in  Table 1. The publication dates ranged from 2003 to 2012. The study sample sizes ranged from 49 to 486 individuals and the number of participants positive for *A. actinomycetemcomitans* ranged from 13 to 204 individuals. Participants’ ages ranged from 4 to 82 years. Definition of periodontal disease varied greatly between the studies. Although majority of the studies defined periodontitis based on probing pocket depth (PPD) and/or clinical attachment level (CAL) measurements, their definitions varied in terms of the threshold for the extent and severity of these criteria. Various researches included a control group of periodontally healthy participants. Eight studies evaluated serotypes a-f and four studies examined serotypes a-e.

**Table 1:**
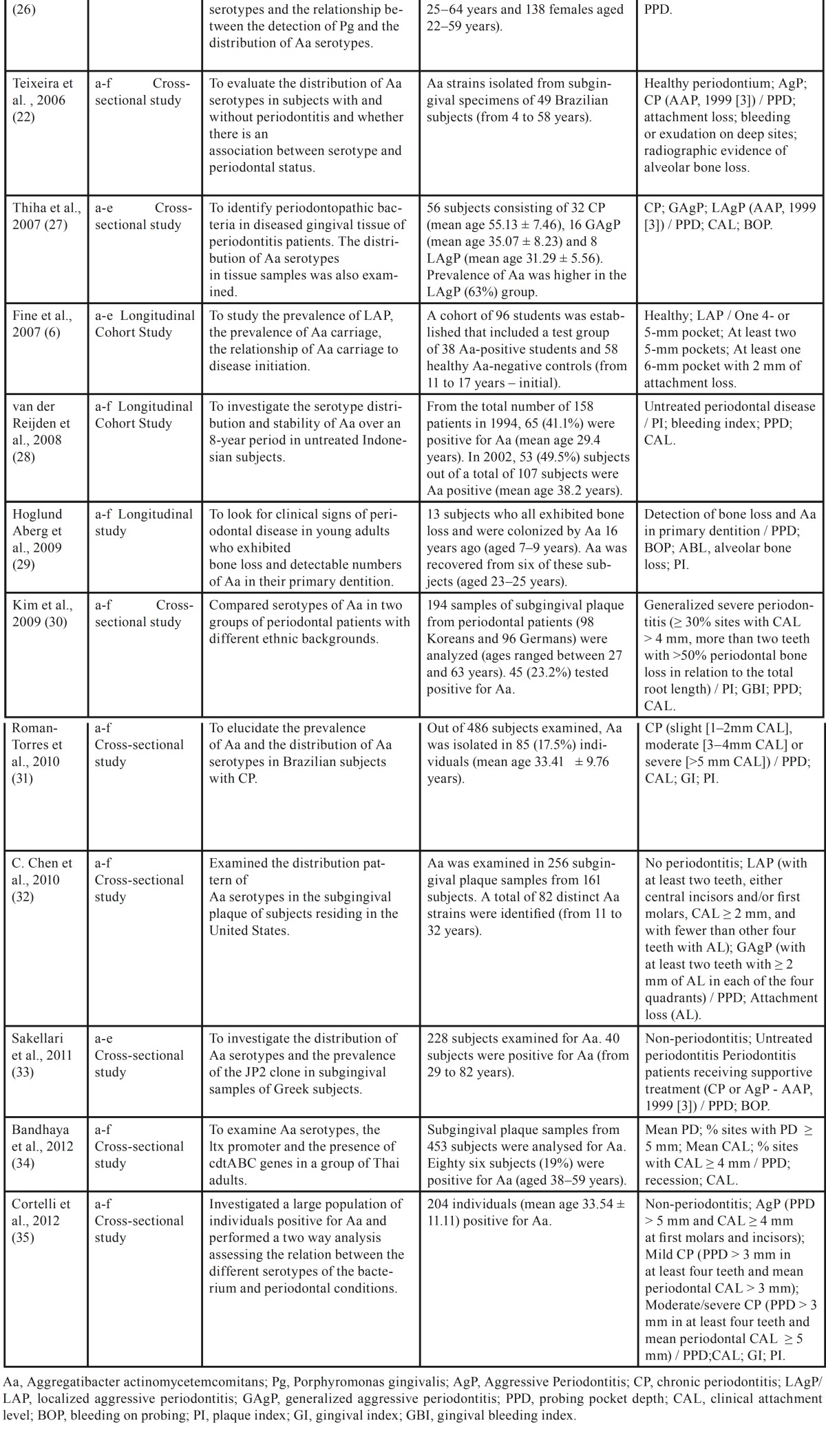
Description of the studies included in the review.

Clinical isolates from diverse geographic populations with different periodontal conditions were evaluated. The samples were obtained of the subjects from Japan, Brazil, United States, Indonesia, Sweden, Germany, Korea, Greece and Thailand.

 Table 2 shows the prevalence and distribution of *A. actinomycetemcomitans* serotypes and the relationship with periodontal status of the studies included in the review. Serotypes a, b and c were largely found, and serotype c was the most prevalent. These serotypes were isolated from various periodontal conditions, including aggressive periodontitis. Serotypes d, e, and f were either not detected or were relatively infrequent.

**Table 2:**
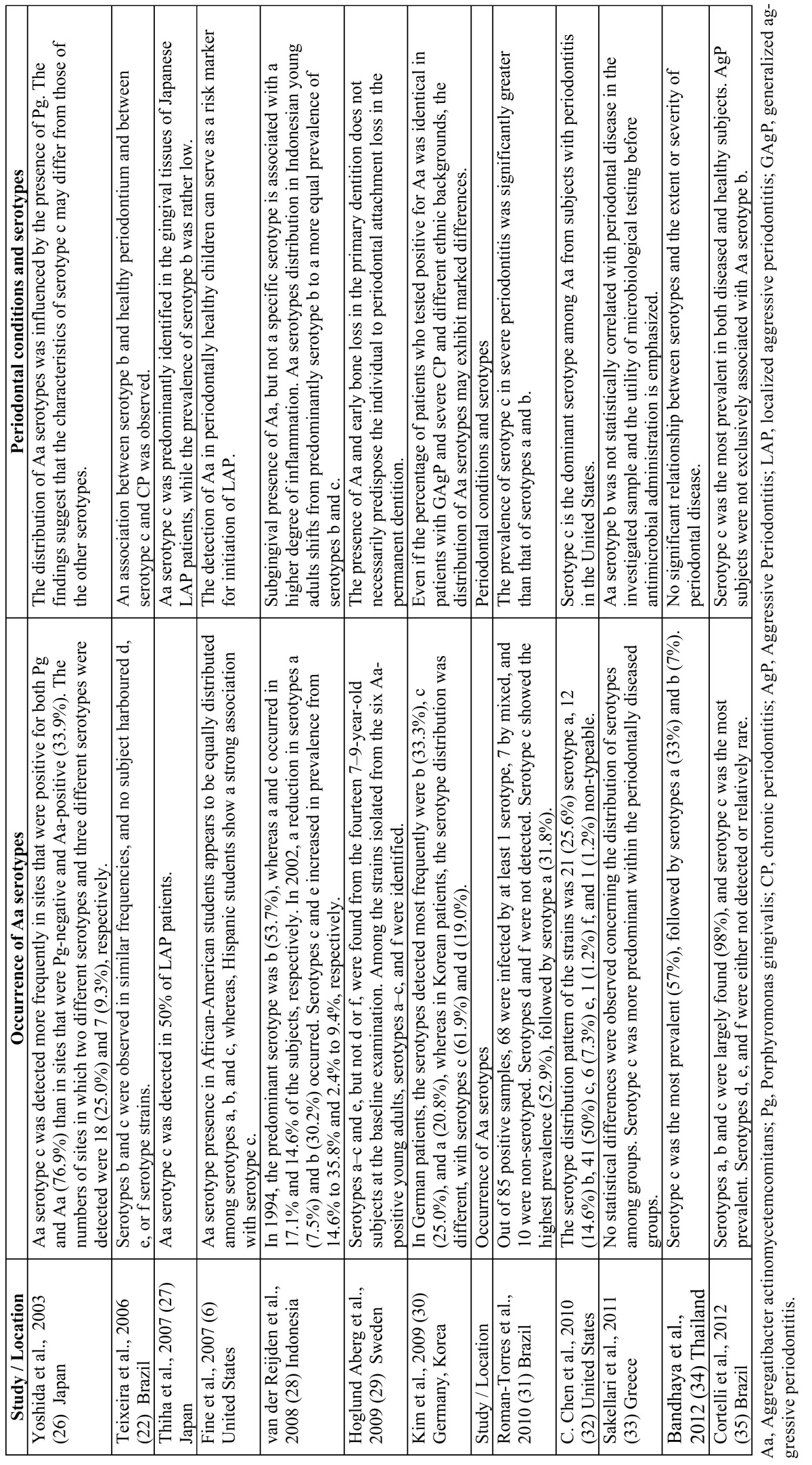
Prevalence and distribution of A. actinomycetemcomitans serotypes and association with periodontal status.

## Discussion

There is convincing evidence of differences in serotype distribution related to geography and/or ethnic group. Available data indicate that the geographic distribution of serotypes is not uniform (6,32,33). The distribution pattern of *A. actinomycetemcomitans* serotypes varies greatly depending on the periodontal status of the allocated population and the country where the study takes place (22,26,27,32,33).

*A. actinomycetemcomitans*, an oral commensal which is also an opportunist pathogen has a distinct racial bias and a surprising range of potential virulence factors and virulence mechanisms. It is a pathogen not only in the periodontium but also in some non oral infections, possesses several virulence determinants which contribute to its ability to colonize the oral cavity, persist in the periodontal pocket, resist and evade host defenses, cause destruction to soft and hard tooth-supporting tissues, and interfere with host tissue repair after infection. Several studies suggest that different *A. actinomycetemcomitans* serotypes are associated with periodontal health, periodontitis, and non-oral infections (13,16,24), therefore the authors reviewed commensal with pathological *A. actinomycetemcomitans*.

It has been suggested that patients are usually infected by only one serotype and colonization is stable over time (28,30), however occasional individuals are colonized with two or three serotypes (31,33,35). Most authors reported frequencies of multipleserotype infection up to 20% (22,27,31). There have been a few exceptions, as in a Japanese study population where two or three serotypes of *A. actinomycetemcomitans* were detected in a percentage as high as 33% of the sites tested positive (26).

In general, the serotypes a-c occur much more frequently among oral isolates than serotypes d-f. The *A. actinomycetemcomitans* serotype presence in African-American students appears to be equally distributed among serotypes a, b, and c, whereas, Hispanic students show a strong association with serotype c (6). Serotypes a, b and c are equally dominant and collectively comprise 95% or more of all *A. actinomycetemcomitans* strains in Greece (33). In Brazilian subjects, serotypes a, b and c were largely found (98%), and serotype c was the most prevalent. Serotypes d, e, and f were either not detected or relatively rare (22,31,35). The distribution pattern of *A. actinomycetemcomitans* serotypes in the subgingival plaque of subjects residing in the United States showed that serotype c is the dominant serotype, followed by serotypes a and b, and serotypes d, e, and f were either not detected or relatively rare (32).

The studies showed that Asian populations were commonly colonized with *A. actinomycetemcomitans* serotype c, but were occasionally infected with serotype b (26,27,30,34). Two studies have examined the serotype distribution patterns of*A. actinomycetemcomitans* in a Japanese population. In both studies serotypes c was the dominant serotype, while serotype b was relatively rare (26,27). For 86 *A. actinomycetemcomitans* strains in Thai adults with varying degrees of periodontal disease severity the serotype c was the dominant serotype, followed by serotypes a (33%) and b (7%) (34), whereas in Korean patients, the serotype distribution was different, the serotypes detected most frequently were c (61.9%) and d (19.0%) (30). The differences between the results from these Asian populations shows that serotype distribution patterns may be affected by geographic variations, even between subjects of the same race/ethnicity.

In contrast, serotype b was frequently observed in Caucasian populations (30). In German patients, the serotypes detected most frequently were b (33.3%), c (25.0%), and a (20.8%) (30).

The serotype distribution pattern of *A. actinomycetemcomitans* within a local population may change over time, as was documented in Indonesian subjects with periodontitis between 1994 and 2002. In 1994, the predominant serotype was b (53.7%), whereas a and c occurred in 17.1% and 14.6% of the subjects, respectively. In 2002, a reduction in serotypes a (7.5%) and b (30.2%) occurred. Serotypes c and e increased in prevalence from 14.6% to 35.8% and 2.4% to 9.4%, respectively (28).

Serotypes d-f were rarely detected in most populations worldwide (32,33,35). However, a high prevalence of serotype e (19-47%) was noted in Indonesian (28) and Japanese (26) individuals.

The application of molecular techniques has allowed a more detailed discrimination among different serotypes of *A. actinomycetemcomitans* and therefore the investigation of potential differences between populations of various origins as well as periodontal conditions. It has been suggested that some *A. actinomycetemcomitans* serotypes are more closely associated with periodontal disease than others.

In the United States, serotype c was the dominant serotype among *A. actinomycetemcomitans* from subjects with periodontitis (32), and in addition to the JP2 serotype b phenotype, there are other strains that are equally associated with disease initiation (6). In Japanese patients, *A. actinomycetemcomitans* serotype c was predominantly identified in the gingival tissues of localized aggressive periodontitis patients, while the prevalence of serotype b was rather low (27), and the distribution of *A. actinomycetemcomitans* serotypes was influenced by the presence of Porphyromonas gingivalis (26).

In Indonesian subjects was observed that the mean increase in probing pocket depth between 1994 and 2002 was significantly greater in subjects culture positive in 2002 in comparison to subjects without detectable *A. actinomycetemcomitans* in 2002 (28). This confirms that subgingival presence of *A. actinomycetemcomitans*, but not a specific serotype is associated with a higher degree of inflammation (6).

In Brazil, an association between serotype b and healthy periodontium and between serotype c and chronic periodontitis was observed (22), differing from other data, which associated serotype b strains with patients with aggressive periodontitis (35). Aggressive periodontitis subjects were not exclusively associated with *A. actinomycetemcomitans* serotype b (31,35). In general, isolates from healthy subjects belonged to serotypes a or c (35). Serotype c was the most prevalent serotype among Brazilian *A. actinomycetemcomitans*, and they were isolated from various periodontal conditions, including aggressive periodontitis (35).

In a Greek population, *A. actinomycetemcomitans* was more prevalent in untreated periodontitis subjects, but no clear predominance of a specific *A. actinomycetemcomitans* serotype and absence of the JP2 clone were observed (33). In Sweden, the findings indicate that periodontitis affecting the primary dentition does not necessarily indicate the presence of periodontal attachment loss in the permanent dentition (29).

Population genetic studies of *A. actinomycetemcomitans* suggest that it is clonal, consisting of genetically distinct subpopulations that correlate with the known serotypes (36,37). It has been proposed that the organism can be grouped into three major phylogenetic lineages comprising serotype b strains, serotype c strains, and serotype a, d, e and f strains (38). Associations between a single serotype b clonal lineage (JP2 clone) and the aggressive form of localized periodontitis in adolescents have been the focus of much investigation (12,39). The JP2 clone shows a limited geographical and ethnic host range, predominating in subjects with an African lineage but absent from non-African populations from Northern Europe (24,40).

The studies have varied widely in periodontal disease diagnosis and status, sampling protocols, study design and microbial detection methods and serotype analysis techniques, hindering comparison of the studies.

The elimination of *A. actinomycetemcomitans* from periodontal pockets has long been considered a target of periodontal therapy, and has been correlated with stable outcomes of treatment. If *A. actinomycetemcomitans* continues to be highly associated with disease deve-lopment, its detection may be used as a risk marker for disease progression.

The findings from the studies reviewed indicate that different ethnic groups are preferentially colonized by different *A. actinomycetemcomitans* serotypes and the relationship between different *A. actinomycetemcomitans* serotypes and periodontal conditions remains unclear.
